# 2-Benzhydryl-6-*tert*-butyl-4-methyl­phenol

**DOI:** 10.1107/S1600536813002006

**Published:** 2013-01-26

**Authors:** Sungwoo Yoon, Junseong Lee, Youngjo Kim

**Affiliations:** a Department of Chemistry, Chungbuk National University, Cheongju, Chungbuk 361-763, Republic of Korea; b Department of Chemistry, Chonnam National University, Gwangju 500-757, Republic of Korea

## Abstract

The title compound, C_24_H_26_O, was prepared by the reaction between 2-*tert*-butyl-4-methyl­phenol and diphenyl­methanol in the presence of sulfuric acid. Three benzene rings are attached directly to the central C—H group in a twisted propeller conformation with the local pseudo-*C*_3_ rotational axis coinciding with the C—H bond. There are three short C—H⋯O contacts in the molecule.

## Related literature

For similar structure types, see: Kim *et al.* (2012 ▶).
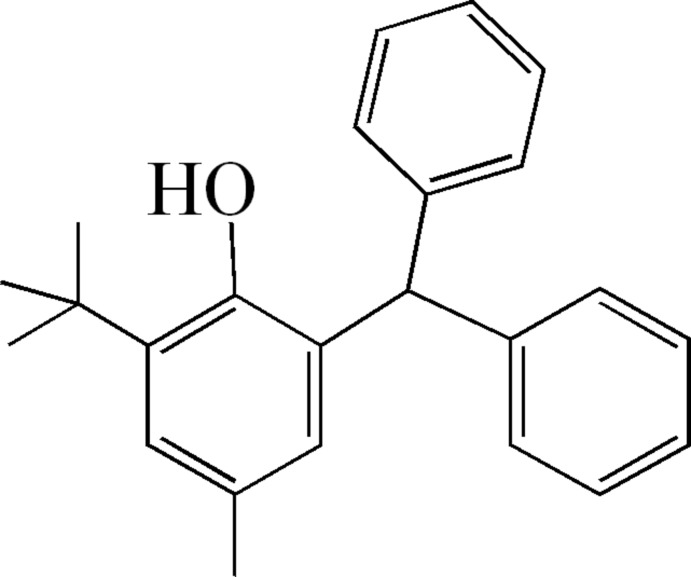


## Experimental

### 

#### Crystal data

C_24_H_26_O
*M* *_r_* = 330.45
Monoclinic, 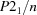

*a* = 8.014 (4) Å
*b* = 15.472 (7) Å
*c* = 16.006 (7) Å
β = 99.98 (2)°
*V* = 1954.6 (15) Å^3^
*Z* = 4
Mo *K*α radiation
μ = 0.07 mm^−1^
*T* = 296 K
0.20 × 0.17 × 0.15 mm

#### Data collection

Bruker APEXII CCD diffractometer
Absorption correction: multi-scan (*SADABS*; Bruker, 2009 ▶) *T*_min_ = 0.987, *T*_max_ = 0.990
17522 measured reflections
3422 independent reflections
1532 reflections with *I* > 2σ(*I*)
*R*_int_ = 0.138

#### Refinement

*R*[*F* ^2^ > 2σ(*F* ^2^)] = 0.071
*wR*(*F* ^2^) = 0.172
*S* = 1.00
3422 reflections
231 parameters
H-atom parameters constrained
Δρ_max_ = 0.15 e Å^−3^
Δρ_min_ = −0.21 e Å^−3^

### 

Data collection: *APEX2* (Bruker, 2009 ▶); cell refinement: *SAINT* (Bruker, 2009 ▶); data reduction: *SAINT*; program(s) used to solve structure: *SHELXS97* (Sheldrick, 2008 ▶); program(s) used to refine structure: *SHELXL97* (Sheldrick, 2008 ▶); molecular graphics: *SHELXTL* (Sheldrick, 2008 ▶); software used to prepare material for publication: *SHELXTL*.

## Supplementary Material

Crystal structure: contains datablock(s) I, global. DOI: 10.1107/S1600536813002006/kj2218sup1.cif

Structure factors: contains datablock(s) I. DOI: 10.1107/S1600536813002006/kj2218Isup2.hkl

Supplementary material file. DOI: 10.1107/S1600536813002006/kj2218Isup3.cdx

Supplementary material file. DOI: 10.1107/S1600536813002006/kj2218Isup4.cml

Additional supplementary materials:  crystallographic information; 3D view; checkCIF report

## Figures and Tables

**Table 1:** Hydrogen-bond geometry (Å, °)

*D*—H⋯*A*	*D*—H	H⋯*A*	*D*⋯*A*	*D*—H⋯*A*
C8—H8*C*⋯O	0.96	2.41	3.049 (4)	123
C10—H10*A*⋯O	0.96	2.44	3.071 (5)	123
C12—H12⋯O	0.98	2.38	2.771 (4)	103

**Fig. 1.**
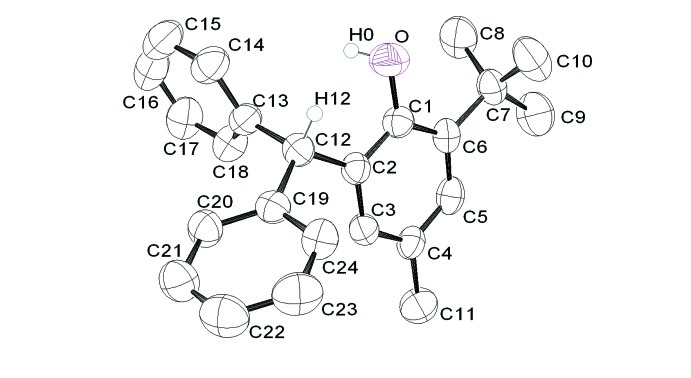
Molecular structure of the title compound, with the atom-numbering scheme. Displacement ellipsoids are drawn at the 50% probability level. H atoms, with exception of H12 and H0, are omitted for clarity.

## supplementary crystallographic information

### Comment

The molecular structure of the title compound, C_24_H_26_O, is shown in Figure 1. The C—O and C—C bond lengths in the phenol ring and two phenyl rings are in the range of typical values determined on similar compounds (Kim *et al.* 2012). The three aromatic rings are twisted and form a propeller conformation with the local pseudo-*C_3_* rotational axis coinciding with C12—H12. The orientation of the rings can be characterized by the torsion angles H12-C12-C2-C1 (-35 °), H12-C12-C19-C24 (-59 °) and H12-C12-C13-C14 (-26 °). The molecules display three intramolecular C—H···O contacts (geometric details are given in Table 1), but no intermolecular hydrogen bonds are present.

### Experimental

The title compound could be isolated in 94% yield *via* the reaction of sulfuric acid, 2-*tert*-butyl-4-methylphenol, and diphenylmethanol in glacial acetic acid. The crystal was obtained by slow evaporation of solvent in refrigerator.

### Refinement

The H-atoms were included in calculated positions and treated as riding atoms with with C—H = 0.93–0.98 Å and O—H = 0.82 Å: *U*_iso_(H) = 1.2 *U*_eq_(parent C-atom),*U*_iso_(H) = 1.5 *U*_eq_(parent O-atom). The initial position of the hydroxyl H was derived from an electron density calculation.

### Crystal data

**Table:**

C_24_H_26_O	*F*(000) = 712
*M**_r_* = 330.45	*D*_x_ = 1.123 Mg m^−^^3^
Monoclinic, *P*2_1_/*n*	Mo *K*α radiation, λ = 0.71073 Å
*a* = 8.014 (4) Å	Cell parameters from 925 reflections
*b* = 15.472 (7) Å	θ = 2.7–16.4°
*c* = 16.006 (7) Å	µ = 0.07 mm^−^^1^
β = 99.98 (2)°	*T* = 296 K
*V* = 1954.6 (15) Å^3^	Block, colorless
*Z* = 4	0.20 × 0.17 × 0.15 mm

### Data collection

**Table:**

Bruker APEXII CCD diffractometer	3422 independent reflections
Radiation source: fine-focus sealed tube	1532 reflections with *I* > 2σ(*I*)
Graphite monochromator	*R*_int_ = 0.138
φ and ω scans	θ_max_ = 25.0°, θ_min_ = 2.6°
Absorption correction: multi-scan (*SADABS*; Bruker, 2009)	*h* = −9→9
*T*_min_ = 0.987, *T*_max_ = 0.990	*k* = −17→17
17522 measured reflections	*l* = −18→19

### Refinement

**Table:**

Refinement on *F*^2^	Primary atom site location: structure-invariant direct methods
Least-squares matrix: full	Secondary atom site location: difference Fourier map
*R*[*F*^2^ > 2σ(*F*^2^)] = 0.071	Hydrogen site location: inferred from neighbouring sites
*wR*(*F*^2^) = 0.172	H-atom parameters constrained
*S* = 1.00	*w* = 1/[σ^2^(*F*_o_^2^) + (0.0803*P*)^2^] where *P* = (*F*_o_^2^ + 2*F*_c_^2^)/3
3422 reflections	(Δ/σ)_max_ < 0.001
231 parameters	Δρ_max_ = 0.15 e Å^−^^3^
0 restraints	Δρ_min_ = −0.21 e Å^−^^3^

### Special details

**Table:**

Geometry. All e.s.d.'s (except the e.s.d. in the dihedral angle between two l.s. planes) are estimated using the full covariance matrix. The cell e.s.d.'s are taken into account individually in the estimation of e.s.d.'s in distances, angles and torsion angles; correlations between e.s.d.'s in cell parameters are only used when they are defined by crystal symmetry. An approximate (isotropic) treatment of cell e.s.d.'s is used for estimating e.s.d.'s involving l.s. planes.
Refinement. Refinement of *F*^2^ against ALL reflections. The weighted *R*-factor *wR* and goodness of fit *S* are based on *F*^2^, conventional *R*-factors *R* are based on *F*, with *F* set to zero for negative *F*^2^. The threshold expression of *F*^2^ > σ(*F*^2^) is used only for calculating *R*-factors(gt) *etc*. and is not relevant to the choice of reflections for refinement. *R*-factors based on *F*^2^ are statistically about twice as large as those based on *F*, and *R*- factors based on ALL data will be even larger.

### Fractional atomic coordinates and isotropic or equivalent isotropic displacement parameters (Å^2^)

**Table:**

	*x*	*y*	*z*	*U*_iso_*/*U*_eq_	
O	−0.0324 (3)	0.35794 (17)	0.04276 (16)	0.0721 (8)	
H0	−0.0789	0.3841	0.0769	0.108*	
C3	0.3942 (4)	0.3867 (2)	0.1619 (2)	0.0473 (9)	
H3	0.4591	0.4304	0.1914	0.057*	
C2	0.2285 (4)	0.4048 (2)	0.1234 (2)	0.0436 (9)	
C13	0.0376 (4)	0.4945 (2)	0.1985 (2)	0.0477 (9)	
C1	0.1322 (4)	0.3377 (2)	0.0801 (2)	0.0459 (9)	
C5	0.3647 (5)	0.2417 (2)	0.1124 (2)	0.0523 (10)	
H5	0.4114	0.1871	0.1088	0.063*	
C4	0.4652 (4)	0.3057 (2)	0.1578 (2)	0.0480 (9)	
C6	0.1977 (4)	0.2555 (2)	0.0721 (2)	0.0473 (9)	
C24	0.3685 (4)	0.5791 (2)	0.0708 (2)	0.0551 (10)	
H24	0.3627	0.5374	0.0285	0.066*	
C19	0.2722 (4)	0.5693 (2)	0.1352 (2)	0.0442 (9)	
C12	0.1469 (4)	0.4931 (2)	0.1297 (2)	0.0449 (9)	
H12	0.0684	0.5009	0.0761	0.054*	
C21	0.3905 (5)	0.7026 (2)	0.1965 (3)	0.0645 (11)	
H21	0.3976	0.7441	0.2389	0.077*	
C23	0.4725 (5)	0.6503 (3)	0.0696 (3)	0.0688 (12)	
H23	0.5354	0.6564	0.0263	0.083*	
C20	0.2870 (4)	0.6314 (2)	0.1981 (2)	0.0535 (10)	
H20	0.2263	0.6252	0.2422	0.064*	
C14	−0.1169 (5)	0.5383 (2)	0.1838 (3)	0.0633 (11)	
H14	−0.1529	0.5650	0.1317	0.076*	
C7	0.0933 (5)	0.1835 (2)	0.0202 (2)	0.0565 (10)	
C18	0.0871 (5)	0.4564 (2)	0.2772 (2)	0.0593 (10)	
H18	0.1895	0.4267	0.2882	0.071*	
C17	−0.0125 (6)	0.4612 (3)	0.3404 (3)	0.0739 (12)	
H17	0.0227	0.4351	0.3928	0.089*	
C22	0.4836 (5)	0.7122 (3)	0.1319 (3)	0.0708 (12)	
H22	0.5530	0.7602	0.1306	0.085*	
C8	−0.0686 (5)	0.1625 (2)	0.0564 (3)	0.0857 (14)	
H8A	−0.0378	0.1443	0.1144	0.129*	
H8B	−0.1298	0.1170	0.0238	0.129*	
H8C	−0.1386	0.2131	0.0537	0.129*	
C9	0.1948 (5)	0.0992 (2)	0.0221 (3)	0.0924 (15)	
H9A	0.2963	0.1094	−0.0006	0.139*	
H9B	0.1274	0.0562	−0.0115	0.139*	
H9C	0.2240	0.0791	0.0795	0.139*	
C15	−0.2170 (5)	0.5426 (3)	0.2458 (3)	0.0772 (13)	
H15	−0.3209	0.5708	0.2347	0.093*	
C16	−0.1636 (6)	0.5053 (3)	0.3238 (3)	0.0807 (14)	
H16	−0.2302	0.5099	0.3657	0.097*	
C10	0.0469 (6)	0.2111 (3)	−0.0734 (2)	0.0864 (14)	
H10A	−0.0214	0.2624	−0.0775	0.130*	
H10B	−0.0155	0.1655	−0.1056	0.130*	
H10C	0.1485	0.2225	−0.0956	0.130*	
C11	0.6475 (5)	0.2879 (2)	0.1987 (2)	0.0733 (12)	
H11A	0.7193	0.2921	0.1567	0.110*	
H11B	0.6558	0.2308	0.2227	0.110*	
H11C	0.6828	0.3295	0.2427	0.110*	

### Atomic displacement parameters (Å^2^)

**Table:**

	*U*^11^	*U*^22^	*U*^33^	*U*^12^	*U*^13^	*U*^23^
O	0.0540 (17)	0.071 (2)	0.086 (2)	0.0068 (14)	−0.0039 (15)	−0.0090 (16)
C3	0.049 (2)	0.048 (2)	0.044 (2)	0.0002 (18)	0.0064 (18)	−0.0042 (18)
C2	0.047 (2)	0.040 (2)	0.043 (2)	0.0048 (18)	0.0068 (17)	−0.0015 (18)
C13	0.044 (2)	0.037 (2)	0.064 (3)	−0.0031 (17)	0.014 (2)	0.0000 (19)
C1	0.047 (2)	0.043 (2)	0.047 (2)	0.0051 (19)	0.0068 (18)	0.0031 (19)
C5	0.071 (3)	0.037 (2)	0.052 (2)	0.009 (2)	0.021 (2)	0.0022 (19)
C4	0.051 (2)	0.048 (2)	0.046 (2)	0.013 (2)	0.0101 (18)	−0.0005 (19)
C6	0.060 (3)	0.041 (2)	0.044 (2)	0.0000 (19)	0.0153 (19)	−0.0031 (19)
C24	0.060 (2)	0.053 (3)	0.054 (2)	0.003 (2)	0.015 (2)	0.000 (2)
C19	0.043 (2)	0.040 (2)	0.047 (2)	0.0047 (17)	0.0011 (18)	0.0055 (19)
C12	0.049 (2)	0.038 (2)	0.047 (2)	0.0041 (17)	0.0030 (17)	0.0017 (17)
C21	0.068 (3)	0.048 (3)	0.077 (3)	−0.005 (2)	0.011 (2)	−0.005 (2)
C23	0.066 (3)	0.072 (3)	0.071 (3)	−0.013 (2)	0.021 (2)	0.019 (3)
C20	0.057 (2)	0.045 (2)	0.060 (2)	−0.0050 (19)	0.0153 (19)	−0.004 (2)
C14	0.055 (2)	0.051 (3)	0.086 (3)	0.003 (2)	0.019 (2)	0.003 (2)
C7	0.068 (3)	0.047 (3)	0.054 (3)	−0.007 (2)	0.013 (2)	−0.009 (2)
C18	0.063 (2)	0.055 (3)	0.062 (3)	0.006 (2)	0.019 (2)	0.008 (2)
C17	0.088 (3)	0.062 (3)	0.079 (3)	−0.004 (2)	0.036 (3)	0.005 (2)
C22	0.070 (3)	0.056 (3)	0.084 (3)	−0.012 (2)	0.009 (3)	0.009 (3)
C8	0.091 (3)	0.071 (3)	0.102 (3)	−0.032 (3)	0.037 (3)	−0.019 (3)
C9	0.108 (4)	0.047 (3)	0.121 (4)	−0.003 (3)	0.018 (3)	−0.027 (3)
C15	0.058 (3)	0.063 (3)	0.116 (4)	0.008 (2)	0.031 (3)	0.001 (3)
C16	0.083 (3)	0.059 (3)	0.112 (4)	0.001 (3)	0.050 (3)	−0.006 (3)
C10	0.104 (4)	0.089 (3)	0.063 (3)	−0.025 (3)	0.009 (3)	−0.018 (3)
C11	0.059 (3)	0.075 (3)	0.081 (3)	0.018 (2)	0.000 (2)	−0.002 (2)

### Geometric parameters (Å, º)

**Table:**

O—C1	1.386 (4)	C20—H20	0.9300
O—H0	0.8200	C14—C15	1.382 (5)
C3—C4	1.382 (4)	C14—H14	0.9300
C3—C2	1.392 (4)	C7—C9	1.535 (5)
C3—H3	0.9300	C7—C10	1.541 (5)
C2—C1	1.403 (4)	C7—C8	1.545 (5)
C2—C12	1.526 (4)	C18—C17	1.395 (5)
C13—C18	1.386 (4)	C18—H18	0.9300
C13—C14	1.395 (4)	C17—C16	1.375 (5)
C13—C12	1.521 (4)	C17—H17	0.9300
C1—C6	1.390 (4)	C22—H22	0.9300
C5—C6	1.398 (4)	C8—H8A	0.9600
C5—C4	1.398 (5)	C8—H8B	0.9600
C5—H5	0.9300	C8—H8C	0.9600
C4—C11	1.519 (5)	C9—H9A	0.9600
C6—C7	1.546 (5)	C9—H9B	0.9600
C24—C23	1.384 (5)	C9—H9C	0.9600
C24—C19	1.400 (4)	C15—C16	1.374 (5)
C24—H24	0.9300	C15—H15	0.9300
C19—C20	1.382 (4)	C16—H16	0.9300
C19—C12	1.540 (4)	C10—H10A	0.9600
C12—H12	0.9800	C10—H10B	0.9600
C21—C20	1.381 (5)	C10—H10C	0.9600
C21—C22	1.384 (5)	C11—H11A	0.9600
C21—H21	0.9300	C11—H11B	0.9600
C23—C22	1.373 (5)	C11—H11C	0.9600
C23—H23	0.9300		
			
C1—O—H0	109.5	C9—C7—C10	107.0 (3)
C4—C3—C2	122.0 (3)	C9—C7—C8	106.9 (3)
C4—C3—H3	119.0	C10—C7—C8	110.3 (3)
C2—C3—H3	119.0	C9—C7—C6	111.5 (3)
C3—C2—C1	118.1 (3)	C10—C7—C6	109.9 (3)
C3—C2—C12	122.5 (3)	C8—C7—C6	111.2 (3)
C1—C2—C12	119.3 (3)	C13—C18—C17	121.8 (4)
C18—C13—C14	117.8 (3)	C13—C18—H18	119.1
C18—C13—C12	122.8 (3)	C17—C18—H18	119.1
C14—C13—C12	119.4 (3)	C16—C17—C18	118.8 (4)
O—C1—C6	120.9 (3)	C16—C17—H17	120.6
O—C1—C2	116.5 (3)	C18—C17—H17	120.6
C6—C1—C2	122.6 (3)	C23—C22—C21	119.4 (4)
C6—C5—C4	123.4 (3)	C23—C22—H22	120.3
C6—C5—H5	118.3	C21—C22—H22	120.3
C4—C5—H5	118.3	C7—C8—H8A	109.5
C3—C4—C5	117.5 (3)	C7—C8—H8B	109.5
C3—C4—C11	121.1 (3)	H8A—C8—H8B	109.5
C5—C4—C11	121.3 (3)	C7—C8—H8C	109.5
C1—C6—C5	116.4 (3)	H8A—C8—H8C	109.5
C1—C6—C7	122.1 (3)	H8B—C8—H8C	109.5
C5—C6—C7	121.6 (3)	C7—C9—H9A	109.5
C23—C24—C19	120.4 (4)	C7—C9—H9B	109.5
C23—C24—H24	119.8	H9A—C9—H9B	109.5
C19—C24—H24	119.8	C7—C9—H9C	109.5
C20—C19—C24	118.2 (3)	H9A—C9—H9C	109.5
C20—C19—C12	123.2 (3)	H9B—C9—H9C	109.5
C24—C19—C12	118.5 (3)	C16—C15—C14	120.3 (4)
C13—C12—C2	111.6 (3)	C16—C15—H15	119.8
C13—C12—C19	113.5 (3)	C14—C15—H15	119.8
C2—C12—C19	114.0 (3)	C15—C16—C17	120.6 (4)
C13—C12—H12	105.6	C15—C16—H16	119.7
C2—C12—H12	105.6	C17—C16—H16	119.7
C19—C12—H12	105.6	C7—C10—H10A	109.5
C20—C21—C22	120.2 (4)	C7—C10—H10B	109.5
C20—C21—H21	119.9	H10A—C10—H10B	109.5
C22—C21—H21	119.9	C7—C10—H10C	109.5
C22—C23—C24	120.6 (4)	H10A—C10—H10C	109.5
C22—C23—H23	119.7	H10B—C10—H10C	109.5
C24—C23—H23	119.7	C4—C11—H11A	109.5
C21—C20—C19	121.2 (4)	C4—C11—H11B	109.5
C21—C20—H20	119.4	H11A—C11—H11B	109.5
C19—C20—H20	119.4	C4—C11—H11C	109.5
C15—C14—C13	120.7 (4)	H11A—C11—H11C	109.5
C15—C14—H14	119.6	H11B—C11—H11C	109.5
C13—C14—H14	119.6		

### Hydrogen-bond geometry (Å, º)

**Table:**

*D*—H···*A*	*D*—H	H···*A*	*D*···*A*	*D*—H···*A*
C8—H8*C*···O	0.96	2.41	3.049 (4)	123
C10—H10*A*···O	0.96	2.44	3.071 (5)	123
C12—H12···O	0.98	2.38	2.771 (4)	103

## References

[bb1] Bruker (2009). *SMART*, *SAINT* and *SADABS* Bruker AXS Inc., Madison, Wisconsin, USA.

[bb2] Kim, S. H., Yoon, S., Mun, S.-D., Lee, H.-H., Lee, J. & Kim, Y. (2012). *Polyhedron*, **31**, 665–670.

[bb3] Sheldrick, G. M. (2008). *Acta Cryst.* A**64**, 112–122. 10.1107/S0108767307043930 18156677

